# Supplementary material for: The interactome of intact mitochondria by cross-linking mass spectrometry provides evidence for coexisting respiratory supercomplexes
Source: Mol Cell Proteomics. 2017 Dec 8;17(2):216–32. doi: 10.1074/mcp.RA117.000470 (PMC5795388; doi:10.1074/mcp.RA117.000470)
Supplement: Supplemental Data [file supp_RA117.000470_133922_0_supp_23978_fzffwf.zip › spectra_annotation/mito_DR_spectra_annotation/315-1-1-1-99-1.pdf]

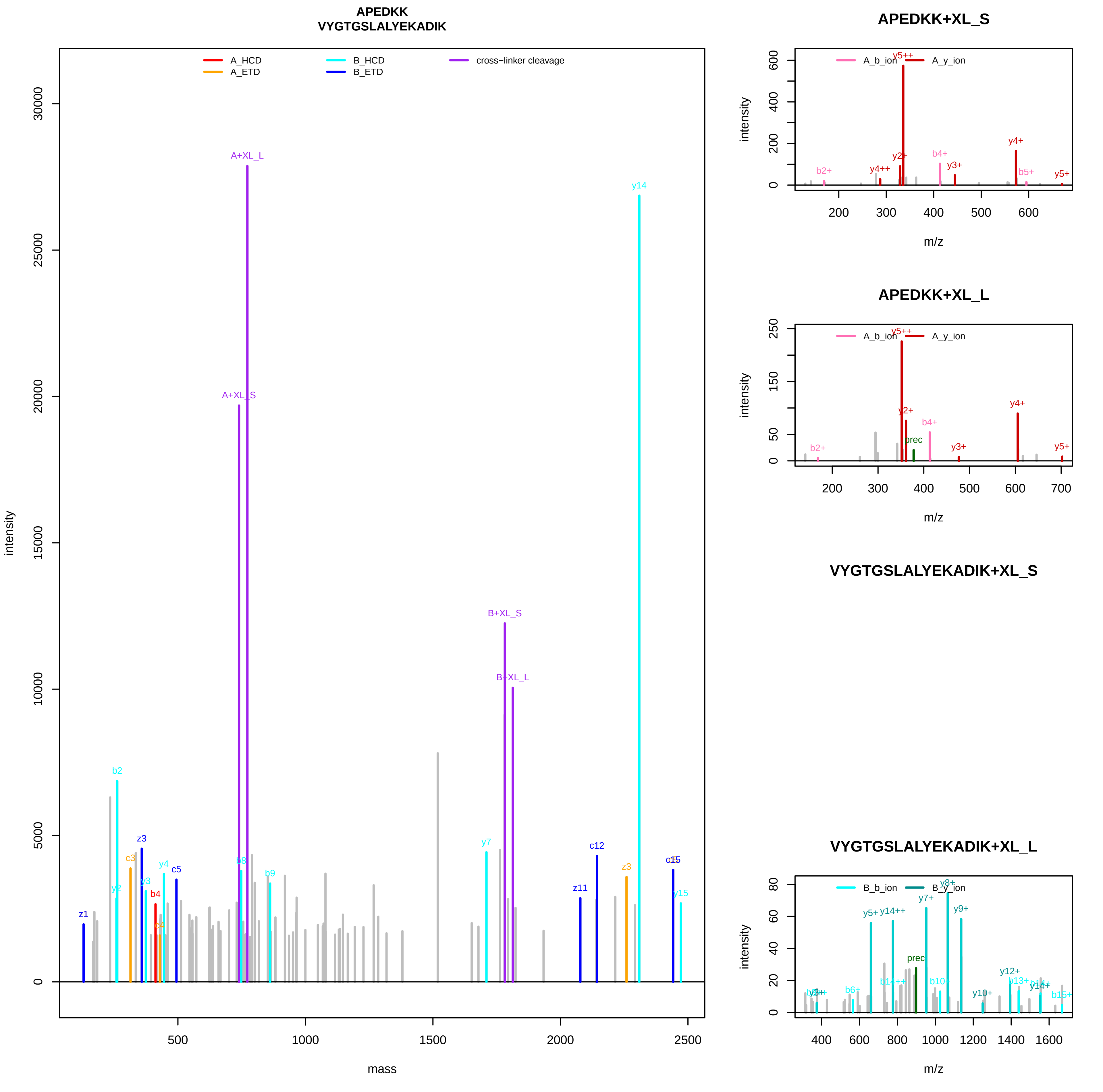

APEDKK
VYGTGSLALYEKADIK
A_HCD
A_ETD
B_HCD
B_ETD
cross−linker cleavage
A+XL_L
A+XL_S
B+XL_S
B+XL_L
y14
b2
z3
c3
y3
y4
c5
b4
c4
z1
y2
b8
b9
y7
c12
z11
z3
c15
y15
intensity
mass
APEDKK+XL_S
A_b_ion
A_y_ion
y5++
y4+
y2+
b4+
y3+
y4++
b2+
b5+
y5+
m/z
APEDKK+XL_L
prec
VYGTGSLALYEKADIK+XL_S
VYGTGSLALYEKADIK+XL_L
B_b_ion
B_y_ion
y8+
y7+
y9+
y5+
y14++
b14++
b10+
b6+
y3+
y12+
b13+
y14+
y10+
b15+
